# *catena*-Poly[[chloridocopper(I)]-μ-η^2^,σ^1^-3-(2-allyl-2*H*-tetra­zol-5-yl)pyridine]

**DOI:** 10.1107/S1600536808013895

**Published:** 2008-06-07

**Authors:** Wei Wang

**Affiliations:** a Ordered Matter Science Research Center, Southeast University, Nanjing 210096, People’s Republic of China

## Abstract

The title compound, [CuCl(C_9_H_9_N_5_)]_*n*_, prepared by solvo­thermal synthesis, is a new homometallic Cu^I^–olefin coordination polymer in which the Cu^I^ atoms are linked by the 3-(2-allyl-2*H*-tetra­zol-5-yl)pyridine ligands and are each bonded to one terminal Cl atom. The organic ligand acts as a bidentate ligand bridging two neighboring Cu centers through the bonds to the N atom of the pyridine ring and the double bond of the allyl group. Weak Cu⋯Cl [3.136 (8) Å), C—H⋯Cl and C—H⋯N inter­actions connect the coordination polymers into a three-dimensional structure.

## Related literature

For the solvothermal synthesis and for related structures, see: Ye *et al.* (2005 ▶,2007 ▶); Wang (2008 ▶).
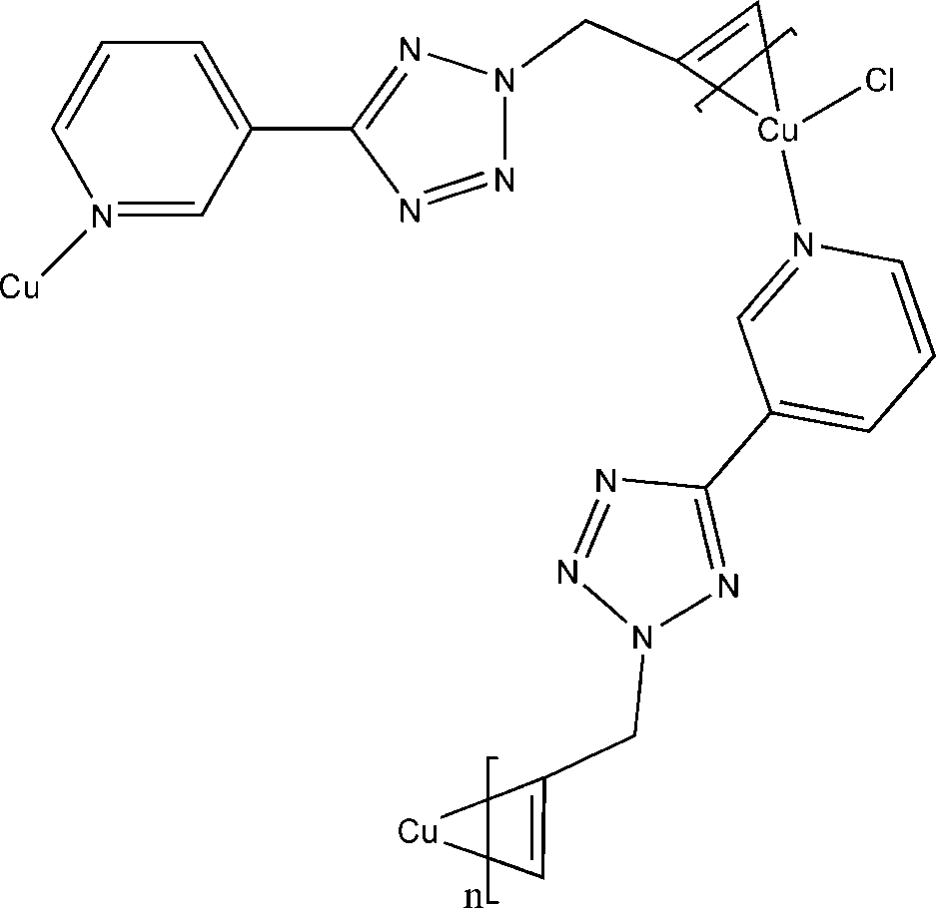


## Experimental

### 

#### Crystal data

[CuCl(C_9_H_9_N_5_)]
*M* *_r_* = 286.21
Triclinic, 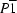

*a* = 7.3005 (15) Å
*b* = 7.6560 (15) Å
*c* = 9.981 (2) Å
α = 80.51 (3)°
β = 77.00 (3)°
γ = 84.68 (3)°
*V* = 535.23 (19) Å^3^
*Z* = 2
Mo *K*α radiation
μ = 2.27 mm^−1^
*T* = 293 (2) K
0.2 × 0.15 × 0.1 mm

#### Data collection

Rigaku Mercury2 diffractometer
Absorption correction: multi-scan (*CrystalClear*; Rigaku, 2005 ▶) *T*_min_ = 0.806, *T*_max_ = 1.000 (expected range = 0.643–0.797)
5572 measured reflections
2443 independent reflections
1918 reflections with *I* > 2σ(*I*)
*R*_int_ = 0.047

#### Refinement

*R*[*F* ^2^ > 2σ(*F* ^2^)] = 0.045
*wR*(*F* ^2^) = 0.103
*S* = 1.16
2443 reflections
154 parameters
H-atom parameters constrained
Δρ_max_ = 0.43 e Å^−3^
Δρ_min_ = −0.46 e Å^−3^

### 

Data collection: *CrystalClear* (Rigaku, 2005 ▶); cell refinement: *CrystalClear*; data reduction: *CrystalClear*; program(s) used to solve structure: *SHELXS97* (Sheldrick, 2008 ▶); program(s) used to refine structure: *SHELXL97* (Sheldrick, 2008 ▶); molecular graphics: *PLATON* (Spek, 2003 ▶) and *SHELXTL* (Sheldrick, 2008 ▶); software used to prepare material for publication: *SHELXTL*.

## Supplementary Material

Crystal structure: contains datablocks I, global. DOI: 10.1107/S1600536808013895/gk2142sup1.cif

Structure factors: contains datablocks I. DOI: 10.1107/S1600536808013895/gk2142Isup2.hkl

Additional supplementary materials:  crystallographic information; 3D view; checkCIF report

## Figures and Tables

**Table:** 

Cu1—N1	1.995 (3)
Cu1—C9^i^	2.026 (3)
Cu1—C8^i^	2.044 (3)
Cu1—Cl3	2.2408 (10)

**Table:** 

N1—Cu1—C9^i^	105.86 (13)
N1—Cu1—C8^i^	143.90 (13)
C9^i^—Cu1—C8^i^	39.16 (14)
N1—Cu1—Cl3	108.44 (9)
C9^i^—Cu1—Cl3	145.70 (11)
C8^i^—Cu1—Cl3	106.88 (10)

Symmetry code: (i) 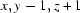.

**Table 2:** Hydrogen-bond geometry (Å, °)

*D*—H⋯*A*	*D*—H	H⋯*A*	*D*⋯*A*	*D*—H⋯*A*
C1—H1*A*⋯Cl3^ii^	0.96	2.79	3.675 (4)	154
C2—H2*A*⋯N4^ii^	0.96	2.59	3.379 (5)	139
C4—H4*A*⋯N4	0.96	2.57	2.909 (4)	101
C6—H6*A*⋯Cl3^iii^	0.96	2.83	3.607 (4)	139

Symmetry codes: (ii) 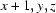; (iii) 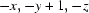.

**Fig. 1.**
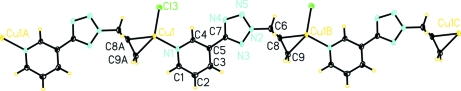
A view of the title compound with displacement ellipsoids shown at the 30% probability level [symmetry codes: A: x, y - 1, z + 1; B: x, y + 1, z - 1; C: x, y + 2, z - 2.

**Fig. 2.**
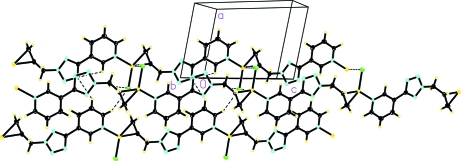
Crystal packing of the title compound viewed along the b axis. Weak interactions are shown as dashed lines.

## supplementary crystallographic information

### Comment

Under hydrothermal or solvothermal conditions some interesting reactions occur. Often new compounds can be obtained that cannot  be synthesized using conventional solution techniques. In sealed tube, unstable copper(I) salt can exist under vacuum, and thus interesting copper(I) coordination compounds can be obtained (Ye *et al.*, 2005, 2007). The title compound, as colorless block crystals suitable for X-ray analysis, was obtained through solvothermal treatment of CuCl and 3-(2-allyl-2*H*-tetrazol -5-yl)pyridine in methanol at 75°C. Isostructural product was obtained when CuBr was used for the reaction (Wang, 2008).

The 3-(2-allyl-2*H*-tetrazol-5-yl) pyridine ligands bind to the copper(I) centers through the N atom of pyridine and double bond of the allyl group (C8—C9 1.364 (5) Å). The copper atom is coordinated to two olefinic organic ligands and one terminal Cl atom in a trigonal environment (Fig 1, Table 1). The organic ligands link the neighboring Cu centers to form a homometallic Cu(I) coordination polymer developing along the *c* axis. Unfortunately, the N atoms of the tetrazole ring fail to coordinate to Cu(I)(Fig. 1).

Finally, weak Cu—Cl (3.136 Å), C–H···Cl and C–H···N interactions between the coordination polymers lead to the formation of the three-dimensional structure (Fig. 2).

### Experimental

A mixture of 3-(2-allyl-2*H*-tetrazol-5-yl)pyridine(20 mg, 0.2 mmol), CuCl (17.9 mg, 0.2 mmol) were placed in a thick Pyrex tube (ca 20 cm in length). After addition of methanol, the tube was frozen with liquid nitrogen, evacuated under vaccum, sealed with a torch and kept at 348 K. Colorless block-shaped crystals suitable for X-ray analysis were obtained after 5 d (yield 61% based on the organic ligand).

### Refinement

All H atoms were fixed geometrically and treated as riding with C—H = 0.93 Å (aromatic), 0.97 Å (methylene) and 0.96Å (methyl) with *U*_iso_(H) = 1.2*U*_eq_(Caromatic, Cmethylene) and *U*_iso_(H) = 1.5*U*_eq_(Cmethyl).

### Crystal data

**Table:**

[CuCl(C_9_H_9_N_5_)]	*Z* = 2
*M**_r_* = 286.21	*F*(000) = 288
Triclinic, *P*1	*D*_x_ = 1.776 Mg m^−^^3^
Hall symbol: -P 1	Mo *K*α radiation, λ = 0.71073 Å
*a* = 7.3005 (15) Å	Cell parameters from 5070 reflections
*b* = 7.6560 (15) Å	θ = 3.2–27.5°
*c* = 9.981 (2) Å	µ = 2.27 mm^−^^1^
α = 80.51 (3)°	*T* = 293 K
β = 77.00 (3)°	Block, colorless
γ = 84.68 (3)°	0.2 × 0.15 × 0.1 mm
*V* = 535.23 (19) Å^3^	

### Data collection

**Table:**

Rigaku Mercury2 diffractometer	2443 independent reflections
Radiation source: fine-focus sealed tube	1918 reflections with *I* > 2σ(*I*)
graphite	*R*_int_ = 0.047
Detector resolution: 13.6612 pixels mm^-1^	θ_max_ = 27.5°, θ_min_ = 3.2°
CCD_Profile_fitting scans	*h* = −9→9
Absorption correction: multi-scan (*CrystalClear*; Rigaku, 2005)	*k* = −9→9
*T*_min_ = 0.806, *T*_max_ = 1	*l* = −12→12
5572 measured reflections	

### Refinement

**Table:**

Refinement on *F*^2^	Primary atom site location: structure-invariant direct methods
Least-squares matrix: full	Secondary atom site location: difference Fourier map
*R*[*F*^2^ > 2σ(*F*^2^)] = 0.045	Hydrogen site location: inferred from neighbouring sites
*wR*(*F*^2^) = 0.104	H-atom parameters constrained
*S* = 1.16	*w* = 1/[σ^2^(*F*_o_^2^) + (0.0388*P*)^2^] where *P* = (*F*_o_^2^ + 2*F*_c_^2^)/3
2443 reflections	(Δ/σ)_max_ < 0.001
154 parameters	Δρ_max_ = 0.43 e Å^−^^3^
0 restraints	Δρ_min_ = −0.46 e Å^−^^3^

### Special details

**Table:**

Geometry. All e.s.d.'s (except the e.s.d. in the dihedral angle between two l.s. planes) are estimated using the full covariance matrix. The cell e.s.d.'s are taken into account individually in the estimation of e.s.d.'s in distances, angles and torsion angles; correlations between e.s.d.'s in cell parameters are only used when they are defined by crystal symmetry. An approximate (isotropic) treatment of cell e.s.d.'s is used for estimating e.s.d.'s involving l.s. planes.
Refinement. Refinement of *F*^2^ against ALL reflections. The weighted *R*-factor *wR* and goodness of fit *S* are based on *F*^2^, conventional *R*-factors *R* are based on *F*, with *F* set to zero for negative *F*^2^. The threshold expression of *F*^2^ > σ(*F*^2^) is used only for calculating *R*-factors(gt) *etc*. and is not relevant to the choice of reflections for refinement. *R*-factors based on *F*^2^ are statistically about twice as large as those based on *F*, and *R*- factors based on ALL data will be even larger.

### Fractional atomic coordinates and isotropic or equivalent isotropic displacement parameters (Å^2^)

**Table:**

	*x*	*y*	*z*	*U*_iso_*/*U*_eq_	
Cu1	0.15988 (6)	−0.16243 (6)	0.38989 (4)	0.03499 (17)	
Cl3	−0.15148 (11)	−0.11291 (11)	0.40805 (10)	0.0362 (2)	
N1	0.2905 (4)	0.0083 (4)	0.2319 (3)	0.0273 (6)	
N2	0.0888 (4)	0.4924 (4)	−0.2268 (3)	0.0288 (6)	
N3	0.2403 (4)	0.4402 (4)	−0.1738 (3)	0.0307 (7)	
N4	−0.0077 (4)	0.2863 (4)	−0.0651 (3)	0.0365 (7)	
N5	−0.0620 (4)	0.4030 (4)	−0.1640 (3)	0.0363 (7)	
C1	0.4677 (5)	0.0501 (5)	0.2253 (3)	0.0345 (8)	
H1A	0.5306	−0.0055	0.2974	0.039 (10)*	
C2	0.5620 (5)	0.1711 (5)	0.1194 (4)	0.0375 (9)	
H2A	0.6894	0.1957	0.1169	0.034 (10)*	
C3	0.4697 (5)	0.2565 (5)	0.0182 (4)	0.0344 (8)	
H3A	0.5327	0.3399	−0.0568	0.040 (10)*	
C4	0.2031 (5)	0.0904 (4)	0.1323 (3)	0.0266 (7)	
H4A	0.0779	0.0591	0.1349	0.029 (9)*	
C5	0.2858 (4)	0.2174 (4)	0.0256 (3)	0.0256 (7)	
C6	0.0802 (5)	0.6439 (4)	−0.3365 (3)	0.0307 (8)	
H6A	0.1222	0.7458	−0.3100	0.030 (10)*	
H6B	−0.0483	0.6696	−0.3450	0.037 (10)*	
C7	0.1745 (5)	0.3120 (4)	−0.0734 (3)	0.0266 (7)	
C8	0.1990 (5)	0.6128 (4)	−0.4751 (3)	0.0303 (8)	
H8A	0.1838	0.5028	−0.5045	0.028 (9)*	
C9	0.3666 (5)	0.6878 (5)	−0.5325 (4)	0.0414 (10)	
H9A	0.4217	0.7393	−0.4705	0.075 (16)*	
H9B	0.4567	0.6248	−0.5960	0.066 (14)*	

### Atomic displacement parameters (Å^2^)

**Table:**

	*U*^11^	*U*^22^	*U*^33^	*U*^12^	*U*^13^	*U*^23^
Cu1	0.0274 (3)	0.0380 (3)	0.0312 (3)	−0.00009 (18)	−0.00526 (18)	0.01645 (19)
Cl3	0.0272 (5)	0.0346 (5)	0.0449 (5)	−0.0015 (3)	−0.0117 (4)	0.0051 (4)
N1	0.0277 (15)	0.0261 (14)	0.0234 (13)	−0.0003 (11)	−0.0041 (12)	0.0069 (11)
N2	0.0307 (15)	0.0278 (15)	0.0243 (14)	−0.0005 (12)	−0.0062 (12)	0.0058 (12)
N3	0.0346 (16)	0.0295 (15)	0.0253 (14)	−0.0053 (12)	−0.0076 (13)	0.0070 (12)
N4	0.0345 (17)	0.0377 (17)	0.0304 (16)	−0.0084 (13)	−0.0051 (13)	0.0160 (14)
N5	0.0321 (17)	0.0388 (18)	0.0335 (16)	−0.0084 (13)	−0.0049 (13)	0.0086 (14)
C1	0.0304 (19)	0.045 (2)	0.0252 (17)	−0.0044 (16)	−0.0086 (15)	0.0090 (16)
C2	0.0280 (19)	0.042 (2)	0.043 (2)	−0.0100 (16)	−0.0110 (16)	0.0004 (18)
C3	0.037 (2)	0.033 (2)	0.0295 (18)	−0.0104 (16)	−0.0036 (16)	0.0069 (16)
C4	0.0246 (17)	0.0270 (17)	0.0235 (16)	−0.0051 (13)	−0.0012 (13)	0.0061 (13)
C5	0.0284 (17)	0.0243 (16)	0.0221 (15)	−0.0020 (13)	−0.0028 (14)	−0.0010 (13)
C6	0.037 (2)	0.0258 (18)	0.0246 (17)	0.0003 (15)	−0.0068 (15)	0.0069 (14)
C7	0.0304 (18)	0.0234 (17)	0.0223 (16)	−0.0028 (14)	−0.0014 (14)	0.0024 (13)
C8	0.036 (2)	0.0221 (17)	0.0275 (17)	0.0038 (14)	−0.0066 (15)	0.0073 (14)
C9	0.0293 (19)	0.041 (2)	0.046 (2)	0.0094 (16)	−0.0110 (18)	0.0140 (18)

### Geometric parameters (Å, °)

**Table:**

Cu1—N1	1.995 (3)	C2—H2A	0.9600
Cu1—C9^i^	2.026 (3)	C3—C5	1.386 (5)
Cu1—C8^i^	2.044 (3)	C3—H3A	0.9600
Cu1—Cl3	2.2408 (10)	C4—C5	1.387 (4)
N1—C4	1.340 (4)	C4—H4A	0.9601
N1—C1	1.345 (4)	C5—C7	1.476 (4)
N2—N5	1.327 (4)	C6—C8	1.501 (5)
N2—N3	1.332 (4)	C6—H6A	0.9600
N2—C6	1.465 (4)	C6—H6B	0.9600
N3—C7	1.321 (4)	C8—C9	1.364 (5)
N4—N5	1.323 (4)	C8—Cu1^ii^	2.044 (3)
N4—C7	1.344 (4)	C8—H8A	0.9600
C1—C2	1.384 (5)	C9—Cu1^ii^	2.026 (3)
C1—H1A	0.9599	C9—H9A	0.9600
C2—C3	1.382 (5)	C9—H9B	0.9600
			
N1—Cu1—C9^i^	105.86 (13)	C5—C4—H4A	118.6
N1—Cu1—C8^i^	143.90 (13)	C3—C5—C4	118.7 (3)
C9^i^—Cu1—C8^i^	39.16 (14)	C3—C5—C7	121.7 (3)
N1—Cu1—Cl3	108.44 (9)	C4—C5—C7	119.6 (3)
C9^i^—Cu1—Cl3	145.70 (11)	N2—C6—C8	113.1 (3)
C8^i^—Cu1—Cl3	106.88 (10)	N2—C6—H6A	108.9
C4—N1—C1	117.8 (3)	C8—C6—H6A	108.7
C4—N1—Cu1	121.4 (2)	N2—C6—H6B	109.0
C1—N1—Cu1	120.7 (2)	C8—C6—H6B	109.1
N5—N2—N3	113.9 (3)	H6A—C6—H6B	107.9
N5—N2—C6	121.6 (3)	N3—C7—N4	112.9 (3)
N3—N2—C6	124.2 (3)	N3—C7—C5	123.1 (3)
C7—N3—N2	101.4 (3)	N4—C7—C5	123.8 (3)
N5—N4—C7	106.3 (3)	C9—C8—C6	123.7 (4)
N4—N5—N2	105.5 (3)	C9—C8—Cu1^ii^	69.73 (19)
N1—C1—C2	122.6 (3)	C6—C8—Cu1^ii^	105.9 (2)
N1—C1—H1A	118.6	C9—C8—H8A	115.7
C2—C1—H1A	118.8	C6—C8—H8A	115.7
C3—C2—C1	119.0 (3)	Cu1^ii^—C8—H8A	116.0
C3—C2—H2A	120.6	C8—C9—Cu1^ii^	71.11 (19)
C1—C2—H2A	120.4	C8—C9—H9A	115.8
C2—C3—C5	118.8 (3)	Cu1^ii^—C9—H9A	116.3
C2—C3—H3A	120.5	C8—C9—H9B	117.2
C5—C3—H3A	120.7	Cu1^ii^—C9—H9B	116.7
N1—C4—C5	123.0 (3)	H9A—C9—H9B	113.5
N1—C4—H4A	118.4		

Symmetry codes: (i) *x*, *y*−1, *z*+1; (ii) *x*, *y*+1, *z*−1.

### Hydrogen-bond geometry (Å, °)

**Table:**

*D*—H···*A*	*D*—H	H···*A*	*D*···*A*	*D*—H···*A*
C1—H1A···Cl3^iii^	0.96	2.79	3.675 (4)	154.
C2—H2A···N4^iii^	0.96	2.59	3.379 (5)	139.
C4—H4A···N4	0.96	2.57	2.909 (4)	101.
C6—H6A···Cl3^iv^	0.96	2.83	3.607 (4)	139.

Symmetry codes: (iii) *x*+1, *y*, *z*; (iv) −*x*, −*y*+1, −*z*.
